# Social media analysis reflects the negative sentiments experienced at both time changes with somewhat more depressive impact in early fall

**DOI:** 10.1371/journal.pone.0342789

**Published:** 2026-03-04

**Authors:** Ben Ellman, Michael L. Smith, Carson Reeling, Nicole J. Olynk Widmar

**Affiliations:** 1 Independent Researcher, Chicago, Illinois, United States of America; 2 Department of Agricultural Economics, Purdue University College of Agriculture, West Lafayette, Indiana, United States of America; University of Lübeck: Universitat zu Lubeck, GERMANY

## Abstract

We quantify the effect of biannual time changes on sentiment using US online and social media posts from periods around changes to Daylight Saving Time (DST) in the spring and Standard Time (ST) in the fall over the period 2019–2023. We compare sentiment—a measure of individuals overall mood or emotions towards an event—in cities on either side of US time zones the day before and after the societal time change. We find negative shocks to sentiment following both time changes. This effect seems stronger in the fall. Given the amount of daylight relative to a fixed work schedule should be the same in each group of cities on these days, these differences suggest strong negative ceteris paribus reactions to societal time changes, which may indicate preference to abolish these adjustments, although we do not measure preference for DST versus ST. We also use regression analysis to estimate how sentiment changes over time. We find persistent negative impacts from the change to Standard Time in the fall. In contrast, individuals experience a noisy shock to sentiment that attenuates over time. These findings provide evidence that individuals have a more negative reaction to the societal time change to Standard Time in the fall than they do to DST in the Spring. This work highlights the potential that the reaction to societal time changes varies depending on whether moving to or away from DST or Standard Time.

## 1. Introduction

Accommodating seasonal changes are a routine part of everyday life. Adapting wardrobes, recreational habits, transportation schedules, and other routines are necessary responses to the new conditions. Aside from temperature changes, the time of sunrise and sunset can be jarring, with massive swings in daylight duration over the course of the year [1]. Longer summer days bring more sunlight than in the winter months. While individuals adapt to these changes in their own way, many countries have adjusted through the controversial implementation of Daylight Saving Time (DST).

In 1916, with World War I in full swing, Great Britain and Germany each passed legislation to push the clocks up an hour in the summer under the theory that the extra sunlight afforded by the new “Summer Time” would lead to energy savings needed for the war effort [2–4]. In 1918, shortly after joining the War, the US followed suit, instituting DST. These policies advanced local time by an hour in late spring/early summer to allocate extra daylight to the evenings [2]. As days naturally grow shorter in the early winter, clocks would fall back to Standard time—the time system used from November to March in the US—to ensure sufficient morning light. The biannual time change was so unpopular in the US, however, that it was repealed after only a year and a half, not to be instituted again until 1942 in response to the US’ involvement in World War II [5]. Like in 1918, the idea that more evening light would conserve fuel through lower lighting demand was enough to convince lawmakers [5].

Following the end of World War II, DST was repealed for a second time, allowing states to establish individual practices surrounding local time and time changes. What followed was a patchwork of policies around the nation, with certain areas observing the time change and others abstaining [2,5]. Industry suffered due to the variability, notably transportation and broadcast segments [2,5]. The divide was especially prominent between rural and urban communities, with many rural areas avoiding DST because of its impact on agriculture schedules while cities adopted the shift to stay in line with financial centers [2]. To abate these disparities, Congress passed the Uniform Time Act in 1966, establishing six months of Standard time and six months of DST, while mandating states to opt in or out of the time change. Arizona, for example, opted out of the measure to avoid prolonging excessive summer heat [2,5].

An attempt to abolish DST occurred in 1974, which quickly became unpopular before the effort was terminated [6]. The biannual societal time change remains broadly unpopular and is frequently, if so far idly, threatened with policy action. Most recently, President Donald Trump made statements condemning DST and promising to use the Republican majority in Congress to eliminate the time change [7].

We seek to measure the change in individual perceptions following semiannual time changes using measures of public sentiment scraped from social media data as a proxy. Sentiment can generally be considered a reflection of the mood or emotions associated with a comment or discussion on a topic. This sentiment can be thought of as a metric gauging the proportion of posts that positively discuss the fall time change away from DST and to Standard time against posts discussing this change in a negative context. We measure the change in sentiment from semiannual time change by comparing estimates of mean sentiment for cities on the west side of each US time zone border on the day of the time change with mean sentiment on the east side the day before (for the change to Standard time) or after (for the change to DST) the time change. We hypothesize (i) that there should be more negative sentiment after the clock shift and (ii) that there will be more negative sentiment in the fall. Absent any effects from the time change, there would be no difference in sentiment estimates between cities on either side of a time zone border; sunrise, and, hence, the amount and timing of daylight relative to a fixed work schedule in a city east (west) of the time zone border on the day of the change to Standard (DST) time should be the same time as in the western (eastern) city the day before the time change. To the extent that sentiment reflects contemporaneous public opinion, sentiment should be the same for both cities. Differences in sentiment would therefore reflect a *ceteris paribus* effect of the time change on individuals sentiment regarding time change events.

We find that time changes have negative contemporaneous impacts on social media sentiment across our sample. A time-series analysis of the sentiment of social media posts during our sampled time frame finds that this negative shock emerges after both time changes, and that this negative shock persists following the change to Standard time in the fall, but attenuates relatively quickly following the switch to DST in spring. This finding aligns with our first two hypothesis. We conclude that the acute periods of societal time changes are negatively associated with sentiment, with the move to Standard Time in the fall being more negatively perceived than the move to DST in the spring.

### 1.2. Prior research on the effects of time changes

The dates on which the shifts to and from DST occur have varied since the passage of the Uniform Time Act, but the practice has continued largely unchanged in the US since. Recent policy measures aimed at ending the time shift have failed in generating required support in Congress, despite large numbers of Americans remaining unhappy with the current biannual shift [4,8]. More than one in five Americans claim the time change has negatively impacted their mental health [9]. Evidence that suicide rates, particularly for males, increase following DST-related time changes strengthen this link [10,11].

The time change affects other aspects of public health and wellbeing as well. People sleep less following the loss of an hour at the start of DST, reducing wellbeing and impairing decision making [12–14]. Additionally, the time change in spring occurs close to the equinox (roughly a week away), resulting in an increase of daylight hours during the waking day for many individuals at the expense of an hour of sleep and disruption of the biological clock [15]. These effects are found with adults and children [16–18]. While there is debate on the topic, it seems road and workplace accidents are more prevalent, as well as the occurrence of certain cardiovascular diseases in the days following the “spring forward” [13,19–21]. There are clear incentives to replace the current system with a permanent alternative, but mapping the benefits and costs of DST and Standard time remains murky.

Part of the indecision over which time regime to make permanent may be the lack of consensus on which permanent solution to adopt. Surveys reveal a majority of Americans claim they would prefer a permanent time solution over seasonal changes, but neither permanent DST nor Standard time garner an outright majority of preference [4,8,9]. Among the reasons for switching to a permanent time, many individuals selected better sleep, alignment of circadian rhythms, and morning safety as important factors to consider, all benefits of moving to permanent Standard time [9,22–29]. However, shifting an hour of daylight from the morning, when many people are asleep, to the evening through DST also held great appeal for many respondents [4]. This could allow more time after work for recreation and consumer spending. There is also the historical suggestion that DST drives down energy usage via reduced demand for lighting. Yet, as daily schedules are less rigid and uniform (such as staggered school start and end times in some geographical regions), the impacts of societal time shifts are likely less obvious and increasingly heterogenous.

Detractors of DST generally favor a switch to permanent Standard time. The American Academy of Sleep Medicine, for instance, favors a permanent switch to Standard time to more closely align social time, or the time the local clock states, with circadian sleep rhythms [9,28]. Jumping ahead an hour under DST exposes individuals to more daylight later in the day. With light being the most important biological cue for the circadian rhythm, this can delay sleep and reduce sleep quality [9,23,25–28]. The negative impacts of DST on sleep can exacerbate negative health outcomes, including obesity, cardiovascular diseases, suicide, and cancer [10,11,20,25]. The increased healthcare costs stemming from overall circadian rhythm disruptions is estimated to exceed $2 billion [25]. Maintaining the natural light cycle with Standard time could bring numerous public health benefits, improving individual outcomes and lowering healthcare expenditures.

Coupled with the externalities associated with public health are concerns that DST affects workplace productivity. There is some evidence that cognitive performance and risk taking are not impaired following the spring DST transition [30]. Analysis of Internet search data, however, shows that non-work-related searches spike following the transition to DST and could often be predicted based on the participant’s sleep duration and quality the previous night [12]. Complex judgment may also be affected by sleep deprivation following the spring transition [14].

Permanent DST would avoid the need for a time change but would still shift daylight later in the day. This would still impact the natural sleep cycle, leading to potential productivity losses for businesses [12,13,25]. Sleep deprivation resulting from misalignment in circadian rhythms with sunlight is estimated to cause 4.4 million days of lost work, equating to $612 million in lost revenue [25].

There are economic costs of DST apparent across industries even beyond lost labor and larger health expenditures. The impact on agriculture is notable when considering the costs of adopting permanent DST or continuing with semi-annual DST. The agriculture industry has been a long-standing opponent of DST since its inception in the US, with the “farm lobby” being a primary driver of the policy’s initial repeal in 1919 [2,31]. Many farmers cite DST’s disruptions to their daily routines, including animal care and crop harvesting. Cows still expect to be milked on a consistent schedule regardless of local time, forcing farmers to deal with unruly livestock or begin their days in the dark to meet adjusted distribution schedules [31,32]. The lost morning light during DST means crops need to be rushed to market to avoid missed shipments, and overall output declines [33]. The agriculture industry remains a staunch detractor of DST, preferring a reliance on natural light instead of clocks to complete essential duties without sacrificing productivity [32].

Though a few groups oppose the expansion and even continuation of DST, many experts point to the policy’s effects on individual wellbeing as reason for it to stay. By allowing access to more light later in the day, DST can enable individuals to spend more time outdoors. More time can be afforded to recreation, evidenced by increases in the physical activity of children during this period [34]. Data from German samples show cycling increases during DST, suggesting that individuals can use the extra light to better their physical fitness [35]. Outdoor activity has been shown to increase by an average of 30 minutes per day under DST, with a 10% increase in calories burned. These health gains alone could save as much as $250 million in annual health expenditures [36]. Yet, the effect of extending DST is not uniformly positive on physical activity as no impact was found in a study of residents in the southwest using an American Time Use Survey [37]. It is possible that heterogeneity in impacts may be due to more localized climates, such as avoiding afternoon heat or due to variations in schedules as work-life integration evolves, albeit heterogeneously. Abolishing DST would reduce the possibility for people to participate in afternoon and evening recreation, potentially contributing to worse health outcomes. Simultaneously, impacts are heterogenous if others gain access to more opportune time for activities or if populations are able to adapt routines, which depends on a wide variety of factors, including available resources for recreation and physical activity.

Aside from exercise, natural light provides other unique health benefits. Vitamin D is an essential nutrient synthesized through exposure to UVB radiation in sunlight. Lower levels of vitamin D are associated with higher rates of cancer, autoimmune disease, cardiovascular disease, and infectious disease [38]. Vitamin D and other benefits attained through increased sun exposure grant natural immunity boosts [38,39]. Given normal work hours, many individuals are unable to access as much sunlight under Standard time, when sunset is an hour earlier in local time. Allowing greater access to daylight during active periods using DST can reduce mortality rates, decreasing cancer deaths in certain populations [39]. Vitamin D deficiency is becoming increasingly widespread, and reduced sun exposure is a key contributor [38]. DST could become an important policy tool in battling this trend and promoting optimal access to sunlight and its many benefits.

Considerations for public safety are another concern when comparing DST and Standard times. Morning and evening commutes are fixed for many with year-round work schedules, making them vulnerable to natural changes in daylight. With darkness being a prime contributor in road accidents, it is important to provide as much light as possible during the most severe accident periods [40]. Previous research has shown that the effect of DST increases the incidence of car accidents both in morning, afternoons, and evenings (despite increased daylight hours) [41]. Fatal car accidents are nearly twice as common during the typical after-work commute than in the morning commute, and evening crashes are even more common relative to the morning in the winter [42].

Though likely driven in part by less overall traffic in the winter, the prevalence of evening accidents may also be due to the loss of cumulative daylight. This is exacerbated by the shift back to Standard time in most states during these months, further reducing evening light. Using DST to allocate an hour of light later in the day would make afternoon and evening driving safer, saving lives and healthcare costs [40]. In addition to positive impacts on public safety on the roads, DST has been shown to reduce crime [29].

Perhaps the foremost argument for DST is its effects on the consumer and the overall economy. Even in its initial implementation in the US, DST was backed by the Chamber of Commerce for its potential to grow evening commerce [2]. The extra light after work would incentivize consumers to spend more at restaurants, entertainment venues, retail stores, and on recreation. Comparisons of consumer purchasing data in areas that observe DST and areas that did not show a positive association between DST and spending, with spending increasing during DST and falling off after [43]. The decrease following the transition from DST had the strongest impact, largely due to less weekday spending [43]. With evening daylight already waning in the winter even without the transition to Standard time, people are more reluctant to purchase goods during the week. Unscheduled shopping trips are also more common during DST [44]. Longer daylight means stores can stay open later, giving customers a chance to make spontaneous purchases after working hours. These purchases are often small, however, so their overall effect on the economy is likely marginal [44].

There is some debate about the net effects of DST on energy use. While lower energy usage has been associated with DST since its inception [3]. More evening daylight should delay the need for artificial lighting, decreasing fuel costs [5]. Energy for lighting is reduced under DST, but overall energy usage is shown to be greater than under Standard time [29,45,46]. Individuals adapt their energy-intensive activities under DST to accommodate these changes [47]. This leads to a higher demand for heating and air conditioning that offsets the savings from lighting, leading to a net increase in energy used. Pressure on electricity markets inflates prices, with the state of Indiana alone estimated to pay as much as $9 million more in annual premiums than under Standard time [45]. The social costs of pollution arising from the higher electricity usage are also significant, with millions being lost to negative externalities such as healthcare costs and natural disasters [45,46]. There is evidence that year-round DST may lead to energy savings during the winter, an alternative to the current policy model [48]. During winter, greater access to daylight when awake may lessen heating demand. Despite this, the environmental costs exacerbated by the current DST model contribute to climate change and rising energy costs.

Other research on the matter of DST has found that preferences are likely to exhibit spatial variance. One study found that preference for seasonal regulation of time is inversely related to latitude [49]. Surveys from Australia before and after seasonal time changes strengthen the idea of attitude towards DST correlating with geographic variation; surveyed individuals further North and West were less likely to prefer DST than those more South and East [50]. This could be a result of lower seasonal variation in the North of Australia, as well as the difference in biological and social time being relatively greater in the West than in the East [50].

Our study builds upon previous literature in two ways. Our primary contribution is to show the usefulness of social media in in measuring interest and sentiment in situations like time changes. We also show an approximately *ceteris paribus* effect of time change on sentiment, proxied by social media mentions and sentiment. To our knowledge, this is the first study to use social media data to attempt to measure perceptions of the change in time regimens which occur in both spring (when we change to DST), and in the fall (when we change to standard time). Secondly, we provide evidence that the negative contemporaneous sentiment, or mood, of time changes (which occur in both spring and fall) are smaller in the spring. These shocks to sentiment abate quickly following time changes in both spring and fall.

## 2. Materials and methods

### 2.1. Social media listening

This paper quantifies social media interest in DST by collecting daily data on social media mentions and sentiment that pertain to the event within a 20-day span surrounding the event. The dataset of mentions was collected using the Quid (formerly Netbase) Social Media Listening platform [51]. The methods used in this paper build upon studies of sentiment of public policy which has previously been investigated in the context of food safety [52,53]. This paper also builds upon previous work in quantifying interest in public goods [54–56].

**Fig 1:**
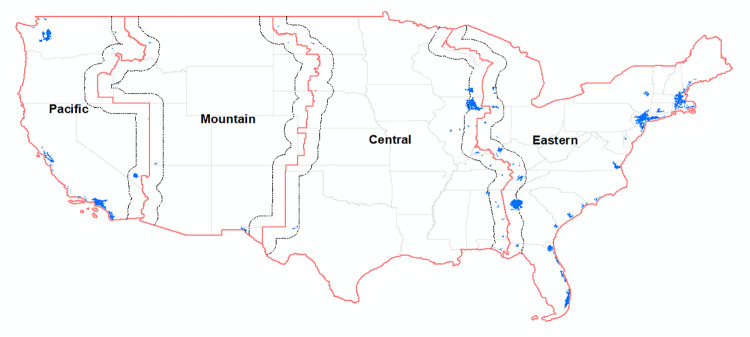
A map of US time zones. Blue regions are cities from which mentions and sentiment data were collected. (Source: author-generated using data from US Census 2024).

We collect online and social media posts based on the following parameters. First, they must match the list of keywords listed in Table 1, which we will refer to as our primary search terms. Primary search terms were determined by a review of articles in popular and published research which discuss DST events. Table 1 also details exclusionary search terms that were used to exclude results from the data collected for the study. Secondly, posts must also originate in specific cities in the USA, shown in Fig 1, and occur on dates listed in Table 2. The cities from which we pull data are located on either side of US time zone borders to facilitate comparison of sentiment across cities that are geographically similar; we describe the logic behind this when discussing our empirical strategy that follows. Social media data was collected by the research team between July 17 and 20, 2023. Due to constraints in social listening on X/Twitter, we can only collect internet data occurring up to 51 months prior to the day data is collected [51]. As such, the period of online data and social media data collection began October 24^th^, 2019. The switch to Standard time that year occurred on November 3, 2019. Data collection efforts complied with the terms and conditions for the sources of the data. Nearly all of our data come from X/Twitter. Only two of a total of 821,140 mentions arose from a different source: mobileread.com, an online chatroom with numerous forums. Online media data on mention counts, sources, and sentiment are available upon request from the corresponding author.

**Table 1:** List of included and excluded terms.

Primary Terms	Excluded Terms
Daylight savings	Peep shit
DST	Jhene Aiko
#DST	Bet My microwave
Spring Forward	Bouquet
Fall Back	Russia
Time change	Russian
Find an hour	
Gain an hour	
Lose an hour	
Extra hour	
Standard time	
Daylight saving	
#Daylightsaving	
#Daylightsavings	
#Timechange	

Note: The first four excluded terms often included in posts containing the phrase “fall back” as these are lyrics in pop music by Jheane Aiko. The remainder reflect exclusion of posts related to the Russian invasion of Ukraine and “fall back” maneuvers undertaken in the context of warfare.

**Table 2:** Dates of data collection.

Year	Spring Dates	Fall Dates
2019	N/A	10/24-11/12
2020	2/27-3/17	10/22-11/10
2021	3/4-3/23	10/28-11/16
2022	3/3-3/22	10/27-11/15
2023	3/2-3/21	10/26-11/14

The Quid platform collects data on posts online and on social media which constitutes a mention of one of the primary terms [51]. By the nature of how various social media and news platforms structure posts, these posts may contain multiple mentions of a primary term. The Quid social media listening platform accommodates this by collecting counts of posts and mentions; as such, the mention count is higher than the post count [51]. Data collected on mentions includes the date of the post. This gives the dataset useful time-varying properties.

These posts are also analyzed by Quid’s Natural Language Processor (NLP) to examine the tone and context of the post. The NLP then assigns each post various attributes, including a sentiment value [51]. Sentiment values can range from –100–100, where positive (negative) values reflect positive (negative) sentiment and a zero reflects neutral sentiment. Quid reports a time varying metric called net sentiment, which reflects the ratio between positive and negative posts on a regular basis [51]. A positive (negative) value of net sentiment indicates a greater share of positive (negative) posts. Both mention counts and net sentiment are observed daily. Net sentiment will be instrumental in evaluating our first two hypotheses, which suggest that, first, there should be more negative sentiment (shown in negative net sentiment) after the clock shift due to the biological clock disruption. Second, we hypothesize that there will be more negative sentiment in the fall because of the depressive effects of increased darkness, which may not yet be attenuated by offsets in morning light. Another feature of Quid’s NLP is its ability to categorize mentions by unique terms that drive the sentiment of each mention of a primary term. The way societies, and individuals, discuss time changes influences how changes are perceived; this is notable and measurable through sentiment analysis. These sentiment drivers are used to analyze how social and online media users feel about DST events and to gauge their reactions in a quantifiable manner.

**Fig 2:**
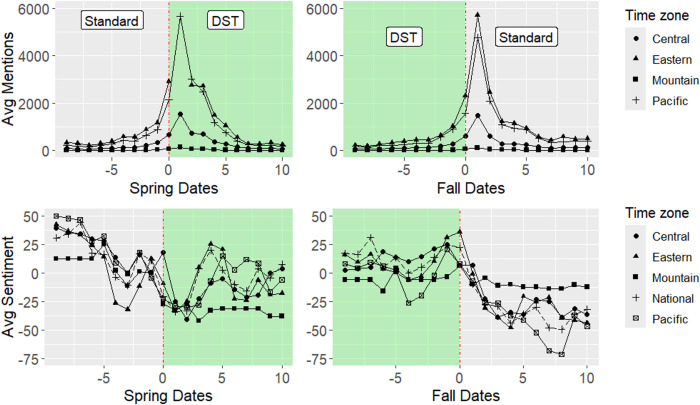
Yearly average daily mentions and sentiment by time zone before and after the time change (day 0).

Fig 2 displays mentions and sentiment by time zone, averaged over all years in our sample. The horizontal axis represents the number of days from each time change, which occurs at time 0. Our dataset includes the time change events and the 20-day span surrounding them. In each case, observations begin 10 days prior to the time change event, which occurs in the morning of day 11, and then observations conclude 9 days after the date of the time change.

### 2.2. Estimating the effects of time changes on individual sentiment and mentions

Given our data, we conduct two analyses to study the effect of the time change on sentiment due to the biannual time change. First, we use regression analysis to estimate a model of how average sentiment changes over time before and after each time change to assess whether shocks to sentiment attenuate over time or lead to secular changes in sentiment. Specifically, we estimate


$$s_{it}=\beta_{\text{0}}+\beta_{\text{1}}t+\beta_{\text{2}}t^{\text{2}}+\beta_{\text{3}}d_{t}+\beta_{\text{4}}d_{t}t+\beta_{\text{5}}d_{t}t^{\text{2}}+\epsilon_{it}$$ (1)

for each biannual time change, where $s_{it}$ is sentiment in region *i* on day *t* ∈ {−10, …, 10}*,* averaged over years, $d_{t\;}=\text{1}(t\geq \text{0})$ is a binary variable equal to one for days after the *t*ime change, and $\epsilon_{it}|t,d_{t}\sim N(\text{0},\sigma )$ is an i.i.d. random shock. The *β* terms are parameters to be estimated.

Second, we estimate an approximate *ceteris paribus* impact of the biannual time change by comparing average sentiment in (i) cities east of each time zone border on the day of the change to Standard time in the fall and (ii) cities west of each time zone border the day before the time change. Our null hypothesis is that there should be no difference in mean sentiment scores between these groups of cities; sunrise (and hence the amount and timing of daylight relative to a fixed work schedule) in an eastern city on the day of the time change (when the clocks “fall back” one hour) should be the same time as in the western city the day before the time change. A statistically significant difference in sentiment across groups of cities (measured via a *t*-test) reveals a *ceteris paribus* effect of the time change on sentiment among individuals.

Likewise, we test whether there is a statistically significant difference between average sentiment in (i) cities east of each time zone border on the day before the change to DST in the spring (when the clocks “spring forward” one hour) and (ii) cities west of each time zone border the day of the change.

## 3. Results

Despite the relatively narrow window of time in which we observe discussion on the topic of Daylight savings in the US, we find that online news and social media are very useful mediums in which to explore public interest in daylight savings, as well as the sentiment (or mood and emotions) associated with these discussions. Each time zone exhibits considerable swings in social media interest leading up to and immediately following each time change. At their peaks the day after the time change, daily mentions for Pacific and Eastern time zones are six times as high as mentions as few as three days prior. In the following days, mentions trail back down to pre-time change levels. This volatility could suggest individuals have a stronger consideration for the effects of the time change itself, rather than the transition to DST vs Standard time. This volatility, and the negative nature of sentiment, may indicate online media users reflect the scientific survey literature that suggests they would support abolishing the changes.

In the national dataset (which combines observations in each region), the mean number of daily mentions of terms in our search parameters during our sample was 32,271 with a standard deviation of 38,019. The national median was 16,739 daily mentions, but national daily mentions range from over 236,000 to as low as 7,357, mirroring the high variability common in the other regions as well. Standard deviation across regions in daily mention counts show a similar trend to the national dataset where we see standard deviation slightly larger than average mentions. The standard deviation is largest in the Eastern (2,995.5) and Pacific (2,645.2) time zones which have the highest average mention counts (2,178.2 and 1,790.5, respectively). Central and Mountain see relatively lower standard deviation in mentions (753.9 and 70.8, respectively), which is intuitive given their lower average counts (547.5 and 45.8, respectively).

Similar to the average daily mentions, average daily sentiment across regions is volatile in the few days before and after each time change. For the national dataset, we observe average daily net sentiment of −3.7 and standard deviation of 33.4. By region, we see a much higher variance. With averages for Eastern, Central, Mountain, and Pacific showing −11.6, −6.1, −21.9, and −17.1. Each of these being negative indicate slightly more negative mentions of DST than positive mentions. Across these regions, the standard deviation on net sentiment is quite high, 73.3 (Eastern), 78.7 (Central), 81.5 (Mountain), and 84.1 (Pacific). There are generally large negative spikes in sentiment immediately leading up to and following a time change, coinciding with the increased interest represented by the trend in mentions. This points to a negative association between time changes and sentiment towards those time changes, strengthening the idea that such shifts are largely disliked. Notably, this dip in sentiment seems to rebound following the spring time change to DST, whereas sentiment remains depressed after the fall change to Standard time.

In the national dataset, the average sentiment under DST (5.65) is greater than under Standard time (−13.02), with the standard deviation for DST being slightly lower than Standard, at 25.32 and 37.71, respectively. This may be caused by seasonal differences. These data should not be interpreted to indicate any slight preference for either time regimen as these are not directly measured. In the spring, we see the sentiments surrounding the change are a little more positive, as both magnitude and consistency of daily sentiment are slightly higher, confirmed via *t*-test (two-tailed p-value of 0.0008).

For robustness, we also test for homogeneity of variances of sentiment and mentions between time change events (DST and Standard) using Levene’s test [57]. Mentions show equivalent variance across groups, but the sentiment test does not (p-value < .001). Given that the sentiment data violates the assumption of homogeneity of variances, a Welch’s t-test is performed, which adjusts for unequal variances across groups; this confirms the relatively higher sentiment in the spring [58]. This could serve as a useful basis for future studies to evaluate whether these differences in sentiment are driven by weather, geography, economics, or other factors while trying to discern if there truly is a preferred time regimen. If policymakers were to abolish time regimen switching, they would need to choose which regimen to adopt. Recent research in the field predicts consequences of the choices regarding abolishing time changes in favor of permanent ST or DST [59].

Table 3 shows parameter estimates for the regression in (1). In spring, there is an insignificant negative time trend prior to the time change at *t* = 0 (i.e., ${\hat{\beta }}_{\text{1}}<\text{0},\;{\hat{\beta }}_{\text{2}}=\text{0}$, where a hat [^] denotes an estimate). A large and negative estimate ${\hat{\beta }}_{\text{3}}$ indicates a decrease in sentiment following the time change, although—consistent with Fig 2—this estimate is noisy. The time trend becomes strongly positive (${\hat{\beta }}_{\text{4}}>\text{0}$) following the time change, suggesting a modest improvement in sentiment. However, the significant and negative quadratic term (${\hat{\beta }}_{\text{5}}$) indicates the change in sentiment following the time change tends to attenuate over time. These results suggest sentiment recovers, consistent with the graphs in Fig 2. In fall, sentiment increases at an increasing rate prior to the change to the time change (${\hat{\beta }}_{\text{1}}>\text{0}$, ${\hat{\beta }}_{\text{2}}>\text{0}$). Following the switch to Standard time, however, the time trend becomes strongly negative (${\hat{\beta }}_{\text{4}}<\text{0},$ ${\hat{\beta }}_{\text{5}}=\text{0}$), suggesting a relatively more persistent decline in sentiment following the time change.

**Table 3:** Regression of sentiment in spring and fall.

Dependent variable	Spring Sentiment		Fall Sentiment	
Explanatory variable	Coefficient	SE	Coefficient	SE
Day relative to time change (*t*)	–3.41	4.36	7.53^**^	3.00
Day squared (*t*^2^)	0.18	0.39	0.61^**^	0.27
Post-time change dummy (*d*)	–19.61	12.57	–34.09^***^	8.64
Interaction terms				
*t* × *d*	11.86^**^	5.67	–15.76^***^	3.90
*t*^2^ × *d*	–0.95^*^	0.55	0.03	0.38
Constant	–12.08	10.45	24.15^***^	7.19
*R* ^2^	0.1127		0.1740	
*N*	720		900	

Superscripts ^*^, ^**^, and ^***^ denote significance at the 10%, 5%, and 1% level, respectively. Note that the post-time change dummy is a binary variable for DST vs ST, with the reference category (0) being the observations just before a time regime changes.

**Fig 3:**
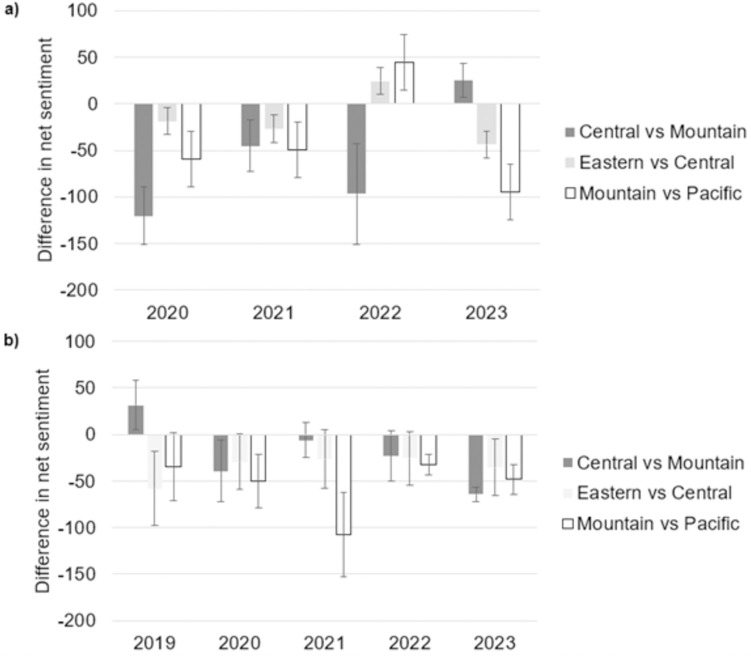
Difference in sentiment by time zone and year in a) cities west of the time zone border on the day after the time change and east of the time zone the day before change to DST in the spring and b) cities east of the time zone border the day after the time change and west of the time zone border on the day before the change to Standard time in the fall. Error bars show 95% confidence intervals.

Fig 3 shows the difference in average sentiment between cities on opposite sides of each time zone the day before and after each time change. Specifically, panel a shows the difference between sentiment in cities west of the time zone border the day after the time change and cities east of the time zone border on the day before the time change to DST in the spring. Panel b shows the difference in sentiment in cities east of the time zone border the day after the time change and west of the time zone border on the day before the change to Standard time in the fall. The effective amount of daylight relative to a fixed work schedule should be the same for each group of cities, and hence a significant negative difference in sentiment would reflect a measure of the change in perceptions from the time change event, holding other environmental factors constant. Fig 3a shows this difference in sentiment is indeed negative and significantly different from zero in most years following the change to DST in spring. Fig 3b shows similar decreases in sentiment, although the decreases are not statistically significant in several of the years.

## 4. Conclusion

By examining social media posts in the US occurring within a 20-day span of DST events between 2020 and 2024, we can measure public interest in the subject and determine the sentiment (or mood and emotions) of discussions online. Both shifts in time regime generate an increase in negative posts relative to positive ones. We find that, both time changes generate negative sentiment on social media which may be stronger in the fall. The negative effects from the spring time change to DST attenuate more quickly, relative to the fall change. The attenuating effect of time on sentiment is similar to the effect of DST on car accidents found in [41]. It is worth noting that the change to DST in the spring precedes warmer weather and longer daylight days whereas the move to Standard time in the fall precedes colder weather and shorter days, which may well explain the differences we observe. These realities are intertwined given the seasonal weather shifts; indeed, seasonal weather patterns as experienced by residents are real and impactful regardless of the local time.

We also find that when time changes occur, the number of mentions on social media are greater in the days after the time change than in the days leading up to it. This indicates that social media users exhibit relatively higher interest in the topic after a time change occurs. Though this interest is largely negative, it strengthens our findings that time changes have measurable effects on people’s sentiment. Any potential policy discussions to amend or replace the current time model will need to consider how alternatives will address short-term negative attention following a change.

There are a variety of limitations which could be addressed or expanded upon in further research. In particular, individuals in different cities on either side of a time zone border may experience different sunrise and sunset times (and therefore daylight hours) due to differences in latitude. The sample of cities we draw from are relatively evenly distributed north to south (Fig 1). The main exception is those cities on the Eastern/Central time zone border; posts from the Central time zone are concentrated in more-northerly Chicago, while posts from Atlanta dominate the Eastern time zone. We expect differences in latitude to impact the magnitude of the decrease in sentiment following time changes but not the direction of the effect. Indeed, focusing just on sentiment in individual time zones (rather than differences in sentiment across time zones), Fig 2 shows that mean sentiment uniformly decreases in each time zone after the time change. Related to this point, cities within the same time zone will experience differences in the distribution of daylight hours due to differences in longitude. We attempt to mitigate this impact by restricting our sample to cities within 100 miles of a time zone border. As before, we expect any bias from differences in the distribution of sunlight hours between cities to affect the magnitude but not the direction of the effect of time changes on sentiment.

We also acknowledge that sentiment towards time changes depends on complex behavioral responses and may be subject to unobserved heterogeneity in sociodemographic characteristics in individuals across space. We attempt to mitigate this by comparing cities that are within 100 miles of a time zone border. We expect that individuals in cities that are relatively close together should exhibit similar characteristics in aggregate. Given the length of the time zones, we acknowledge that there are likely significant differences in the demographics of cities as one travels from north to south along a time zone border. We expect these differences to average out assuming the distribution of cities in the sample is comparable across time zones. Finally, our data span the COVID pandemic, with major shutdowns starting in March 2020. It is reasonable to suspect that changes in sentiment may differ in pre- and post-pandemic shutdowns due to secular changes in activity levels (e.g., working from home versus commuting to work) and in social interactions. We only have one pre-COVID time period in the data (covering the change to Standard time in 2019), but we did compare average sentiment in the fall 2019 by time zone with average sentiment in years 2020–2023. We find no statistically significant difference in these averages and conclude that COVID did not have a meaningful effect on perceptions of time change given our data. In addition to the limitations above, future research could explore whether one time regimen (ST or DST) is preferred, as we do not measure this.

## Supporting information

S1 File Include the minimal data set for analysis. (XLSX)
